# Neurocysticercosis: Neglected but Not Forgotten

**DOI:** 10.1371/journal.pntd.0001500

**Published:** 2012-05-29

**Authors:** Christina M. Coyle, Siddhartha Mahanty, Joseph R. Zunt, Mitchell T. Wallin, Paul T. Cantey, A. Clinton White, Seth E. O'Neal, Jose A. Serpa, Paul M. Southern, Patricia Wilkins, Anne E. McCarthy, Elizabeth S. Higgs, Theodore E. Nash

**Affiliations:** 1 Albert Einstein College of Medicine, Bronx, New York, United States of America; 2 Helminth Immunology Section, Laboratory of Parasitic Diseases, National Institute of Allergy and Infectious Diseases, National Institutes of Health, Bethesda, Maryland, United States of America; 3 Departments of Neurology and Global Health, University of Washington, Seattle, Washington, United States of America; 4 Department of Neurology, Georgetown University, Washington, D.C., United States of America; 5 University of Maryland, Baltimore, Maryland, United States of America; 6 Parasitic Diseases Branch, Centers for Disease Control, Atlanta, Georgia, United States of America; 7 Department of Internal Medicine, Infectious Disease Division, University of Texas, Galveston, Texas, United States of America; 8 Oregon Health & Sciences University, Portland, Oregon, United States of America; 9 Department of Medicine, Section of Infectious Diseases, Baylor College of Medicine, Houston, Texas, United States of America; 10 Pathology and Internal Medicine, University of Texas Southwestern Medical Center at Dallas, Dallas, Texas, United States of America; 11 Department of Medicine, Ottawa General Hospital, Ottawa, Ontario, Canada; 12 Collaborative Clinical Research Branch, Division of Clinical Research, National Institute of Allergy and Infectious Diseases, National Institutes of Health, Bethesda, Maryland, United States of America; 13 Gastrointestinal Parasites Section, Laboratory of Parasitic Diseases, National Institute of Allergy and Infectious Diseases, National Institutes of Health, Bethesda, Maryland, United States of America; World Health Organization, Switzerland

Neurocysticercosis (NCC) is an infection of the central nervous system caused by the larval form of the tapeworm *Taenia solium*. Infections occur following the accidental ingestion of tapeworm ova found in human feces. NCC is a major cause of epilepsy and disability in many of the world's poorer countries where families raise free-roaming pigs that are able to ingest human feces. It is frequently diagnosed in immigrant populations in the United States and Canada, reflecting the high endemicity of the infection in their countries of origin [1]. Although parenchymal cysts are the most common location in the brain and cause seizures, cysts may also be present in the ventricles, meninges, spinal cord, eye, and subarachnoid spaces. Involvement in these other sites may result in aberrant growth (racemose cysts) and complicated disease that is difficult to treat and may cause increased morbidity and mortality.

Until recently, NCC and echinococcosis were referred to as “neglected” diseases, but are now recognized by the World Health Organization (WHO) as “major neglected diseases” [2]. In many endemic communities, NCC is the cause of epilepsy in ≥1% of the population. Here, we provide an update of the importance of NCC, recent epidemiological observations, and an outline of the goals of a recently formed group of physicians, the North American Neurocysticercosis Clinical Research Consortium (NACC).

NCC is difficult to identify and treat. Diagnosis requires brain imaging, a technology commonly unavailable in resource-poor areas, and clinical diagnosis is unreliable, as the manifestations are diverse and non-specific. Additionally, treatment commonly involves weeks to months of medication with close monitoring for side effects and serial brain imaging. NCC is a significant health problem in Latin American countries (LAC), Haiti, sub-Saharan Africa (SSA), India, Southeast Asia, China, Indonesia, and other regions less well categorized, such as New Guinea and Eastern Europe [2]. Although accurate estimates of the extent of infection and disease are not available for most endemic regions, many studies suggest a higher prevalence of infection and disease burden than has been appreciated by experts [3], [4].

Conventional diagnosis of NCC requires a compatible clinical history, positive serology, and typical computed tomography (CT) or magnetic resonance imaging (MRI) [5]. Determination of the prevalence of infection is hampered by variable sensitivity and specificity of each of the diagnostic tests, which identify a limited or overlapping proportion of the “true” prevalence of infection. For instance, the sensitivity of the serum electroimmunotransfer blot decreases dramatically in those with calcified disease or a single parenchymal cyst [6] and may be falsely positive in persons exposed but not infected.

Better estimates of infection can be obtained by imaging of the brain to ensure inclusion of individuals with NCC infection who are asymptomatic and seronegative to *T. solium* (see Box 1). One study in Peru that characterized prevalence of infection and NCC using a combination of CT imaging, positive serology, and history of seizures to identify infections [7] reported an NCC seroprevalence of 24%. An additional 13% of those with negative serology had CT scans demonstrating calcifications typical of NCC. Adding the observed proportion of seropositive individuals to seronegative individuals with CT imaging evidence of NCC yields an overall prevalence of cysticercosis of 37%. Even this substantial proportion of infection likely underestimates its prevalence in this population. Applying similar calculations to earlier comparable studies performed in Ecuador [8], Honduras [9], and Mexico [10] yields prevalence estimates of infection of 23%, 38%, and 15%, respectively. Therefore, estimates of prevalence as defined in LAC range between 15% and 38%. Applying these prevalence calculations to the 75 million persons at risk in LAC found in 1993 [11] we estimate between 11 and 29 million persons have cysticercosis in LAC. Thus, the burden of infection with cysticercosis and NCC is substantial, and likely higher in absolute numbers with the increasing population currently at risk. These numbers also allow a more appropriate comparison between prevalence estimates of other common infections in LAC [3].

**Box 1. Terms Used in This Manuscript**

**Infection:** Any or all of the following: Evidence of prior ingestion of *T. solium* ova resulting in aborted development and positive antibody response, presence of viable or degenerated dead *T. solium* cysts anywhere in the body irrespective of antibody response. Includes a positive serology for antibodies or antigen, calcifications in brain or muscle, or viable or degenerating parasites.

**Seroprevalence:** Usually a defined population testing positive for antibodies to *T. solium* cysts. Less commonly positive for cestode antigen in serum.

**Neurocysticercosis:** Evidence of *T. solium* infection of the brain with or without symptoms.

Our estimate of the prevalence of NCC in LAC is substantially higher than that suggested by Bern et al. [11], which estimated symptomatic NCC only, using fairly conservative assumptions. An estimate of mostly active epilepsy was determined by Bern et al. by subtracting the epilepsy rates in non-endemic regions from rates in endemic regions in Peru [11]. Extrapolating to the 1993 population at risk and adjusting for disease other than seizures, the authors calculated that 400,000 individuals in LAC had symptomatic NCC. More recent studies using direct determinations of active epilepsy due to NCC from active epilepsy rates ranging from 0.6% to 1.8% suggest that between 450,000 and 1.35 million persons suffer from epilepsy due to NCC in LAC (Table 1).

**Table 1:** Estimated Numbers of Persons Infected with Neurocysticercosis or Epilepsy due to Neurocysticercosis by Region.

Geographic Region	Number Infected	Number with NCC^a^	Number with Epilepsy due to NCC^a^
Latin America	11–29 million	14.9 million	0.45–1.35 million
India	-	-	1 million
China	3–7 million	-	0.3–0.7 million^b^
Africa	-	-	0.31–4.6 million^c^

a See text for methods used to derive these estimates.

b Assumes that 10% of those seropositive to *T. solium* have epilepsy. (Extraction of data from manuscripts summarized in references [7], [11], [31]). Seizure rates in populations seropositive to *T. solium* were greater than in seronegative populations by approximately 9.4% (range 1.7–31.3%). Therefore, we assumed 10% of seizures were attributable to NCC in Chinese seropositive populations.

c Assuming 1/3 of the SSA population of 560 million at risk.

Similar to Latin America, many areas of Asia are endemic for NCC. Studies in endemic regions of India reveal high burdens of infection and symptomatic NCC [2]. In a community study in southern India, the seroprevalence of *T. solium* infection was 15.9%, active epilepsy was present in 3.83/1,000 persons, and 28.4% of these individuals had NCC detected by CT imaging. Extrapolating these figures to the overall population in India suggests that approximately 1 million cases of epilepsy are due to NCC [12]. Cysticercosis is also highly endemic in southwest China. According to a recent WHO report, the average prevalence of *T. solium* infection in China was 0.11% (range, 0.05%–15%); the estimated number of patients with taeniasis was 1.26 million; and the estimated number of cysticercosis cases was 3–6 million [2].

Although SSA is among the least studied regions, accumulating evidence suggests highly endemic transmission with significant symptomatic disease. High prevalence in pigs in many areas suggests a high level of human taeniasis [13], [14]. Serologic studies suggest many regions of SSA have endemic infection that likely affects large proportions of the population (Table 2 - adapted from Winkler et al. [4]). Epilepsy prevalence in SSA ranges from 5.2 to 74.4 per 1,000 persons. A recent systematic analysis of the proportion of epilepsy endemic regions due to NCC was estimated at 29% (95% CI: 22.9–35.5) [15]. Using this figure, the prevalence of seizures due to NCC in SSA would be 1.7–24.8 per 1,000 persons.

**Table 2:** Serologic Studies of Human Cysticercosis/Neurocysticercosis in Sub-Saharan Africa.

Region	Population Investigated	Percentage Positive (%)	Reference
Western Africa	PWE (3 studies; *n* [range] = 88–504)	1.2–44.6	[13], [20], [21]
	Non-epileptic controls (6 studies; *n* [range] = 319–5,264)	0.4–40	[20], [22]–[26]
Eastern Africa	PWE (3 studies; *n* [range] = 104–324)	4.9–59.6	[27]
	Patients with other medical issues (*n* = 489)	12.1	[28]
	Non-epileptic controls (3 studies; *n* [range] = 72–648)	2.0–31.5	Refer to [27]
Southern Africa	PWE (*n* = 92)	37.0	[29]
	Patients with neurological disorders (*n* = 630)	12	[30]

PWE, patients with epilepsy.

Reports of NCC in the United States and Canada have also been increasing over the past 50 years, and these reports mostly reflect disease in Hispanic immigrants [1], [16]–[18]. Regional estimates in the general population range from 0.2 to 0.6 per 100,000 in Oregon and Los Angeles [18], [19]. Of those presenting to urban emergency departments because of seizures, 2.1% were attributable to NCC [18], similar to the proportion of seizure patients with NCC (2%) in Houston [16]. This population also presents with complicated subarachnoid disease, or racemose cysticercosis, which is characterized by cysts that proliferate, enlarge, and develop membranes in the subarachnoid spaces of the brain, mostly involving the basilar cisterns and spine. Sequelae include hydrocephalus, arachnoiditis, infarction, and entrapment syndromes requiring long-term corticosteroids, anthelminthics, and serial imaging. These individuals are frequently referred to tertiary care centers where they may be under the care of NACC participants.

Recognizing the importance of NCC in immigrant communities from endemic regions in North America, a group of infectious disease and travel medicine practitioners have come together to focus efforts on management of NCC. The goals of the NACC are to disseminate information regarding diagnosis and treatment of NCC, highlight areas of research need, and advance knowledge through retrospective and prospective collaborative studies of treatment for the more complicated forms of NCC. Initially, we are developing standardized methods to evaluate neuroimaging and morbidity and hope to develop guidelines for treatment.

In summary, recent carefully performed studies of the prevalence and burden of disease have recognized NCC as a major cause of epilepsy in endemic regions and a cause of complicated brain disease in immigrant populations. Resources directed at defining high prevalence regions, preventing infection, and treating epilepsy and other complications, all achievable with present technology, and would reduce the burden of this debilitating disease worldwide.

## References

[1] Wallin MT, Kurtzke JF (2004). Neurocysticercosis in the United States: review of an important emerging infection.. Neurology.

[2] Savioli LS, Daumerie D (2010). First WHO report on neglected tropical diseases: working to overcome the global impact of neglected tropical diseases.

[3] Hotez PJ, Bottazzi ME, Franco-Paredes C, Ault SK, Periago MR (2008). The neglected tropical diseases of Latin America and the Caribbean: a review of disease burden and distribution and a roadmap for control and elimination.. PLoS Negl Trop Dis.

[4] Winkler AS, Willingham AL, Sikasunge CS, Schmutzhard E (2009). Epilepsy and neurocysticercosis in sub-Saharan Africa.. Wien Klin Wochenschr.

[5] Del Brutto OH, Rajshekhar V, White AC, Tsang VC, Nash TE (2001). Proposed diagnostic criteria for neurocysticercosis.. Neurology.

[6] Prabhakaran V, Rajshekhar V, Murrell KD, Oommen A (2004). Taenia solium metacestode glycoproteins as diagnostic antigens for solitary cysticercus granuloma in Indian patients.. Trans R Soc Trop Med Hyg.

[7] Montano SM, Villaran MV, Ylquimiche L, Figueroa JJ, Rodriguez S (2005). Neurocysticercosis: association between seizures, serology, and brain CT in rural Peru.. Neurology.

[8] Del Brutto OH, Santibanez R, Idrovo L, Rodriguez S, Diaz-Calderon E (2005). Epilepsy and neurocysticercosis in Atahualpa: a door-to-door survey in rural coastal Ecuador.. Epilepsia.

[9] Medina MT, Duron RM, Martinez L, Osorio JR, Estrada AL (2005). Prevalence, incidence, and etiology of epilepsies in rural Honduras: the Salama Study.. Epilepsia.

[10] Fleury A, Gomez T, Alvarez I, Meza D, Huerta M (2003). High prevalence of calcified silent neurocysticercosis in a rural village of Mexico.. Neuroepidemiology.

[11] Bern C, Garcia HH, Evans C, Gonzalez AE, Verastegui M (1999). Magnitude of the disease burden from neurocysticercosis in a developing country.. Clin Infect Dis.

[12] Rajshekhar V, Raghava MV, Prabhakaran V, Oommen A, Muliyil J (2006). Active epilepsy as an index of burden of neurocysticercosis in Vellore district, India.. Neurology.

[13] Zoli AP, Nguekam, Shey-Njila O, Nsame Nforninwe D, Speybroeck N (2003). Neurocysticercosis and epilepsy in Cameroon.. Trans R Soc Trop Med Hyg.

[14] Prado-Jean A, Kanobana K, Druet-Cabanac M, Nsengyiumva G, Dorny P (2007). Combined use of an antigen and antibody detection enzyme-linked immunosorbent assay for cysticercosis as tools in an epidemiological study of epilepsy in Burundi.. Trop Med Int Health.

[15] Ndimubanzi PC, Carabin H, Budke CM, Nguyen H, Qian YJ (2010). A systematic review of the frequency of neurocyticercosis with a focus on people with epilepsy.. PLoS Negl Trop Dis.

[16] del la Garza Y, Graviss EA, Daver NG, Gambarin KJ, Shandera WX (2005). Epidemiology of neurocysticercosis in Houston, Texas.. Am J Trop Med Hyg.

[17] Ong S, Talan DA, Moran GJ, Mower W, Newdow M (2002). Neurocysticercosis in radiographically imaged seizure patients in U.S. emergency departments.. Emerg Infect Dis.

[18] Townes JM, Hoffmann CJ, Kohn MA (2004). Neurocysticercosis in Oregon, 1995–2000.. Emerg Infect Dis.

[19] Sorvillo FJ, Waterman SH, Richards FO, Schantz PM (1992). Cysticercosis surveillance: locally acquired and travel-related infections and detection of intestinal tapeworm carriers in Los Angeles County.. Am J Trop Med Hyg.

[20] Dumas M, Grunitzky E, Deniau M, Dabis F, Bouteille B (1989). Epidemiological study of neuro-cysticercosis in northern Togo (West Africa).. Acta Leidensia.

[21] Zoli A, Shey-Njila O, Assana E, Nguekam JP, Dorny P (2003). Regional status, epidemiology and impact of Taenia solium cysticercosis in Western and Central Africa.. Acta Trop.

[22] Avode DG, Bouteille B, Houngbe F, Adjien C, Adjide C (1998). Epilepsy, cysticercosis and neurocysticercosis in Benin.. Eur Neurol.

[23] Houinato D, Ramanankandrasana B, Adjide C, Melaku Z, Josse R (1998). Seroprevalence of cysticercosis in Benin.. Trans R Soc Trop Med Hyg.

[24] Nguekam JP, Zoli AP, Zogo PO, Kamga AC, Speybroeck N (2003). A seroepidemiological study of human cysticercosis in West Cameroon.. Trop Med Int Health.

[25] Kanobana K, Praet N, Kabwe C, Dorny P, Lukanu P (2011). High prevalence of Taenia solium cysticerosis in a village community of Bas-Congo, Democratic Republic of Congo.. Int J Parasitol.

[26] Secka A, Grimm F, Marcotty T, Geysen D, Niang AM (2011). Old focus of cysticercosis in a senegalese village revisited after half a century.. Acta Trop.

[27] Winkler AS, Blocher J, Auer H, Gotwald T, Matuja W (2009). Epilepsy and neurocysticercosis in rural Tanzania-An imaging study.. Epilepsia.

[28] Vilhena M, Santos M, Torgal J (1999). Seroprevalence of human cysticercosis in Maputo, Mozambique.. Am J Trop Med Hyg.

[29] Foyaca-Sibat H, Cowan LD, Carabin H, Targonska I, Anwary MA (2009). Accuracy of serological testing for the diagnosis of prevalent neurocysticercosis in outpatients with epilepsy, Eastern Cape Province, South Africa.. PLoS Negl Trop Dis.

[30] Mason P, Houston S, Gwanzura L (1992). Neurocysticercosis: experience with diagnosis by ELISA serology and computerised tomography in Zimbabwe.. The Central African Journal of Medicine.

[31] Flisser A, Gyorkos TW (2007). Contribution of immunodiagnostic tests to epidemiological/intervention studies of cysticercosis/taeniosis in Mexico.. Parasite Immunol.

